# Time and Motion Analysis of Controlled Substance Disposals: A Study of Workflows at a Single Center using Automated Dispensing Cabinets

**DOI:** 10.1097/ao9.0000000000000006

**Published:** 2026-03-17

**Authors:** Cassandra W. Cu, Timothy Heintz, Nicole Dundas, Hassan Farhan, Hunter Mills, Jennifer W. Lee, Christian Williams, Arthur W. Wallace, Julien Cobert

**Affiliations:** 1 School of Medicine, Tufts University School of Medicine, Boston, Massachusetts.; 2 Department of Anesthesiology, Perioperative, and Pain Medicine, Brigham and Women’s Hospital, Harvard Medical School, Boston, Massachusetts.; 3 Department of Bioengineering, University of California, Berkeley, California.; 4 Department of Anesthesia, El Camino Hospital, Burlingame, California.; 5 Bakar Computational Health Sciences Institute, University of California, San Francisco, California.; 6 Department of Pharmacy, San Francisco Veterans Affairs Medical Center, San Francisco, California.; 7 Division of Anesthesia, San Francisco Veterans Affairs Medical Center, San Francisco, California.

## To the Editor:

Controlled substance chain-of-custody is highly regulated at national and local levels. Controlled substance diversion—transfer of substances from a lawful to unlawful use—remains a persistent problem and anesthesiologists are uniquely vulnerable.^1^ Although the estimated diversion prevalence is 1% to 8%, events are underreported.^2^ Inappropriate tampering has been linked to infections,^3^ and institutions face significant penalties when diversion occurs.^4^ Current prevention practices focus largely on administrative tracking. While automated dispensing cabinets serve as key interfaces for controlled substance security and documentation, evidence for diversion reduction is limited.^1,5^ Despite advances in inventory systems, controlled substance disposals remain a trust-based “honor system” producing workflow friction.^1,6^ Understanding anesthetist interactions with automated dispensing cabinets can identify vulnerabilities and guide improvements to minimize workflow interruptions and enhance accountability.

In this institutional review board (University of California, San Francisco, approved #24-42977) single-center prospective study, we performed a time-and-motion analysis over the course of 6 weeks (May to June 2025), examining controlled substance disposal workflows. This study was performed at a single centralized automated dispensing cabinet (BD Pyxis, Becton Dickinson, USA) at the San Francisco Veterans Affairs Medical Center post-anesthesia care unit (PACU). See Supplemental Digital Content 1 (https://links.lww.com/ALNO/A4) for further details on local practices. All participants consented using institutional review board–approved consent forms. Participants included clinicians who handle controlled substances: certified nurse anesthetists, physician anesthesiologists, anesthesiology resident physicians, and PACU registered nurses (RNs). We followed teams bringing patients to the PACU through controlled substance disposal. We conducted surveys assessing attitudes about diversion and wasting processes. We adhered to Strengthening the Reporting of Observational Studies in Epidemiology (STROBE) guidelines (Supplemental Digital Content 1, https://links.lww.com/ALNO/A4).^7^

Our study was informed by Toyota lean management frameworks and principles of Six Sigma.^8,9^ See Supplemental Digital Content 1 (https://links.lww.com/ALNO/A4) for framework details. Week 1 observation was used to iterate case report forms, which remained unchanged for the remainder of the study (Supplemental Digital Content 1, Table 1, https://links.lww.com/ALNO/A4). We minimized Hawthorne effects by having observers avoid interaction with participants.^10^ We provide a glossary of workflow in Supplemental Digital Content 1, Table 2 (https://links.lww.com/ALNO/A4). We performed descriptive statistics using means, SD, medians, and interquartile ranges when appropriate. Missing subevent observations (*e.g.*, time to recruit a witness) were rare (less than 2%) and, when they occurred, were excluded from analyses. Observations were anonymized and recorded manually.

To assess attitudes and opinions toward controlled substance disposal processes, we conducted anonymous surveys consisting of questions using either 7-point Likert scale (1 = extremely disagree to 7 = extremely agree) or percentages (0% to 100%; Supplemental Digital Content 1, Table 3, https://links.lww.com/ALNO/A4). Surveys assessed satisfaction and trust in controlled substance disposal processes, attitudes around other diversion-detection approaches, and perceptions of usefulness and burden of surveillance practices. We used Excel (v16, USA) for data analysis.

**Fig. 1.**
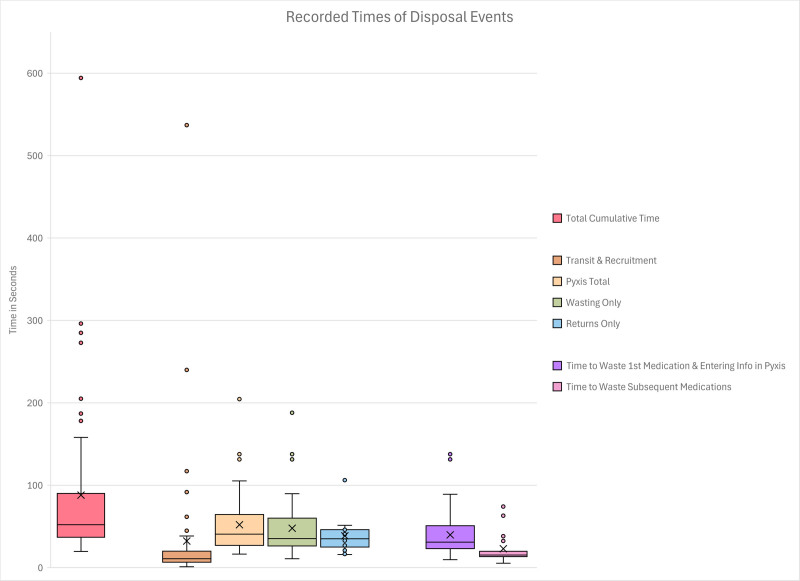
Box and Whisker Plot highlighting various times recorded for CS disposal. “*X*” designates the average, while the *line in the middle* of the box plot represents the median values. The boundaries of each box represent quartile 1 and quartile 3. The *dots* represent outliers affecting the skew of each timed category. Pyxis represents the specific automated dispensing cabinet used at the study site. Total cumulative time is the time from announcement for need to dispose CS until ADC logout (captures transit and recruitment time + pyxis total + wasting only and/or returns only). Transit and recruitment time captures waiting time for an available witness and the physical distance traveled from location of disposal announcement (*e.g.*, patient bedside) to ADC. Pyxis total is the time from clinician login to logout of ADC. Wasting only describes time spent in ADC where only wasting events occurred, while returns only is the time spent in ADC where only returns transpired. Time to waste first medication includes time to log into ADC, time to search patient medication, medication name, calculate dose and volume of CS to be disposed, and ends with time to expel CS into waste bin. Time to waste subsequent medications includes the time to dispose of a second or third medication in a single ADC transaction but excludes the time to log into ADC and search for information and the time to waste the first medication. ADC, automated dispensing cabinet; CS, controlled substances.

Over the study period, 55 disposal events were observed involving 19 anesthesiologists, 16 certified registered nurse anesthetists (CRNAs), 12 PACU nurses, and 8 residents. All individuals approached consented to be part of the study. Most pairings were CRNA-RNs (25.6%), MD-resident MDs (20%), MD-RNs (12.7%), RN-RNs (12.7%), MD-CRNAs (12.7%) or other combinations (16.3% ). No participants withdrew and no events led to discrepancies. See Supplemental Digital Content 1, Table 4 (https://links.lww.com/ALNO/A4) for disposal characteristics. During witness recruitment, 50.9% of witnesses were performing direct patient care, while 49.1% were involved in other tasks like administrative tasks, non-clinical work, electronic health record data entry or indirect patient care (see Supplemental Digital Content 1, Table 4, https://links.lww.com/ALNO/A4). Mean waiting time for a witness was 32.2 s (SD: 81.3 s), ranging from 1 to 537 s. Clinicians followed predictable disposal workflows (Supplemental Digital Content 1, Figure 1, https://links.lww.com/ALNO/A4), although 16.4% of disposals occurred before patient handoff was completed. Table 1 shows data entry patterns and label/volume demonstrations. Mean automated dispensing cabinet time was 52.0s (SD 36.2) ranging 16.4s to 204.6s and represented the longest subcomponent of the disposal process. Figure 1 presents box-and-whisker plots of disposal times. In 80% of events, custodians and not witnesses, entered automated dispensing cabinet data. Syringe labels and volumes were both shown to witnesses in 41.8% of disposals, and neither was shown in 38.2% (Table 1). Supplemental Digital Content 1, Table 5 (https://links.lww.com/ALNO/A4) shows times for process components.

**Table 1.** Proportion of Observed Events Where CS Custodian, the Witness, or Both Entered Data into Pyxis

Who Entered Waste Information into Pyxis and Physically Showed Label *versus* Volume to Witness (Total CS Disposals, N = 55)					
	Label and Volume Shown	Label Shown but not Volume	Label not Shown but Volume Was Shown	Neither Label nor Volume Shown	Total
CS custodian entry only	18 (32.7%)	6 (10.9%)	4 (7.3%)	16 (29.1%)	44 (80%)
Witness entry only	3 (5.5%)	0 (0.0%)	1 (1.8%)	2 (3.6%)	6 (10.9%)
Mixed CS custodian/witness entry	2 (3.6%)	0 (0.0%)	0 (0.0%)	3 (5.5%)	5 (9.1%)
Total	23 (41.8%)	6 (10.9%)	5 (9.1%)	21 (38.2%)	55

For each category, the number of times the label and/or volume was explicitly shown/not shown to the witness was enumerated. All values are rounded to the tenth decimal point.

CS, controlled substances.

Sixty-one surveys were distributed with 28 responses returned (45.9% response rate). Participant demographics are shown in Supplemental Digital Content 1, Table 6 (https://links.lww.com/ALNO/A4). Survey responses are summarized in Supplemental Digital Content 1, Table 7 (https://links.lww.com/ALNO/A4) and Supplemental Digital Content 1, Figure 2 (https://links.lww.com/ALNO/A4). Most respondents (71.5%) felt that diversion events could still occur within existing systems, and many felt that diversion would not be identified (39.3%) or not avoided (32.1%). Many respondents felt that existing disposal processes were inadequate to prevent discrepancies (42.8%). Many felt current processes were labor-intensive and time-consuming. Most respondents believed their colleagues were reading the label (median: 78%, interquartile range: 0.5 to 0.9) and volume of medications (median: 75%, interquartile range: 0.4 to 0.9) during each controlled substance disposal transaction but the range was wide (label: 10% to 100%; volume: 5% to 100%). Most agreed that they would be comfortable with cameras being used to facilitate disposal (67.9% at least somewhat agreed). Many respondents felt there may be high levels of diversion occurring nationally (median: 25%, interquartile range: 5% to 35%). Results are shown in Supplemental Digital Content 1, Table 8, https://links.lww.com/ALNO/A4.

This study highlights important tensions within automated dispensing cabinet–based drug disposals—users perceive disposals to be inefficient, time-consuming, and unreliable in deterring diversion—even though actual observed times are relatively short. Further, disposal accounting is not reliably performed (Table 1), which could explain some of the respondents’ skepticism around these processes. Our respondents estimated a high prevalence of national diversion (approximately 25%), which differs from national estimates and may reflect a lack of trust, locally, in these disposal processes. While this study was not centered around diversion perceptions, further research is needed to better capture perceived *versus* actual diversion rates and whether there may be unrecognized issues of trust and accountability within anesthesia. Despite clinicians perceiving that labels and volumes were demonstrated, few observed disposals showed labels and volumes to witnesses, and concerningly, automated dispensing cabinet data are almost always entered by custodians (80%). These vulnerabilities when aiming to ensure independent verification could undermine the core diversion deterrence purpose of witness-based disposal. Although our centralized automated dispensing cabinet may not reflect other practices, our findings illustrate general trade-offs in efficiency and accountability. Our results suggest that exploring more efficient (*e.g.*, automated dispensing cabinet entry) and dependable workflows (*e.g.*, cameras) are needed to ensure accountable controlled substance disposal. Understanding workflows could guide design improvements in automated dispensing cabinet systems. Our study has important limitations including: brief observation period, narrow focus on disposal interactions limiting generalizability, and extremely small sample size. Another limitation is that the Hawthorne effect may have influenced behavior, including underestimating inappropriate disposals and discrepancy rates.

Notable vulnerabilities exist during witness-based disposals and these processes are felt to be time-consuming and unreliable. Future work should explore alternative solutions to ensure proper and accountable controlled substance disposal.

## Acknowledgments

The authors sincerely thank the clinicians and staff at the San Francisco Veterans Affairs Medical Center Post Anesthesia Care Unit (San Francisco, California) for assisting them with this study.

## Research Support

Support was provided solely from institutional and/or departmental sources.

## Competing Interests

Drs. Cobert and Heintz have a provisional patent application on using artificial intelligence and optical sensors to monitor medication administration and disposals. Drs. Heintz and Wallace are shareholders in Atapir Inc., which is a company building a platform around contact-free computer vision-based clinical tools. The corresponding author and coauthors have not published, posted, or submitted any related papers from the same study to any other print or electronic publications, preprint servers, or other repositories. Neither artificial intelligence nor large language models were used in any aspect of this study.

## Supplemental Digital Content

Supplemental methods, Supplemental Digital Content 1: https://links.lww.com/ALNO/A4.

Case Report Form used for TMA data collection, Supplemental Digital Content 1, Table 1: https://links.lww.com/ALNO/A4.

TMA terms glossary and definitions, Supplemental Digital Content 1, Table 2: https://links.lww.com/ALNO/A4.

Survey questions administered, Supplemental Digital Content 1, Table 3: https://links.lww.com/ALNO/A4.

Characteristics of CS disposal events, Supplemental Digital Content 1, Table 4: https://links.lww.com/ALNO/A4.

All recorded times related to CS disposal, Supplemental Digital Content 1, Table 5: https://links.lww.com/ALNO/A4.

Survey participant demographics, Supplemental Digital Content 1, Table 6: https://links.lww.com/ALNO/A4.

Survey responses, Supplemental Digital Content 1, Table 7: https://links.lww.com/ALNO/A4.

Free response questions and responses, Supplemental Digital Content 1, Table 8: https://links.lww.com/ALNO/A4.

Overall disposal processes at the SFVAMC, Supplemental Digital Content 1, Figure 1: https://links.lww.com/ALNO/A4.

Dual-axis charts of survey responses regarding attitudes toward current disposal processes, monitoring, detection, and CS surveillance, Supplemental Digital Content 1, Figure 2: https://links.lww.com/ALNO/A4.

## Supplementary Material
