# Supplementary material for: Time and Motion Analysis of Controlled Substance Disposals: A Study of Workflows at a Single Center using Automated Dispensing Cabinets
Source: Anesthesiol Open. 2026 Mar 17;1(1):e0006. doi: 10.1097/ao9.0000000000000006 (PMC12998741; doi:10.1097/ao9.0000000000000006)
Supplement: Supplementary file 1 [file ao9-1-e0006-s001.pdf]

Supplemental Methods:

Details on SFVAMC practice, ADC location:

This study was performed at a single centralized ADC (Becton Dickinson [Bd], Pyxis) at the SFVAMC post-anesthesia care unit (PACU). This ADC is under continuous 24-hour security camera surveillance (through the SFVAMC police department; used for review if a criminal incident were to occur) and located within a locked clinical area. The SFVAMC is a mid-sized anesthesia group with 9 main ORs, 4 non-OR anesthesia sites. The practice is a physician anesthesiologist-supervised CRNA-driven practice with rotating student nurse anesthetists and resident anesthesia physicians. Most cases are directly performed by CRNAs.

While out of the scope of this anesthesia workflow study, some drug disposal, discrepancy and diversion practices are performed after clinicians perform ADC-facilitated and witness-based disposal processes. At the SFVAMC, PACU charge nurses and pharmacists perform daily audits of ADCs to identify inventory and disposal discrepancies. When a discrepancy is identified, pharmacists will chart review anesthetic records to identify CS charted and directly contact anesthetists (typically the custodian and the witness) via secure email to address any errors. Pharmacists, themselves, will not audit records unless a discrepancy is identified. A separate CS disposal team within the SF VAMC performs manual audits quarterly of every ADC on a random basis within the institution (>50 ADCs) to monitor for discrepancies not found via Pyxis data entry.

Details on Toyota and Lean frameworks:

The goal of this study was to characterize workflows of anesthesia clinicians during the CS disposal process and how they interact with ADCs. We adopted Toyota production lean

management principles and frameworks to inform our scientific approach to identify future opportunities for minimizing waste and improving workflows.[1,2] The tasks we observed were modeled by the eight major types of non-value-adding activities per Toyota's lean philosophy, including: defects in services, overproduction (e.g. overstaffing, storage, transportation costs), reducing idle time, transportation, overprocessing or incorrect processing, excess inventory, unnecessary movement, and unused clinician talent (i.e. losing time, ideas, skills and learning opportunities).[2] Our methodology also incorporated foundational principles of Six Sigma, aiming to reduce medical errors and decrease defects in medical processes.[2] In order to better understand which of the Toyota principles would be highlighted by current SFVAMC PACU CS disposal practices, we first conducted a 1-week pilot TMA. During this first week of the 6-week study, we performed pilot observations of existing CS disposal processes to inform and develop a TMA case form. This was iterated across 2 members of the research team (Cu, Cobert) and led to a finalized case report form, which remained unchanged for the remainder of the study (see Supplemental Table 1 for case form). After the initial week of observation, one researcher (Cu) formally observed anesthesia clinician workflows for an additional 5 weeks.

Supplement Bibliography

1. Cohen, R. I. Lean Methodology in Health Care. *Chest* **154**, 1448–1454 (2018).
2. Liker, J. K. & Meier, D. *The Toyota Way Fieldbook: A Practical Guide for Implementing Toyota's 4Ps*. (McGraw-Hill, New York, 2006).

Supplemental Tables:

**Participants:**			
**Subject ID (###):**			
**Date (DD/MM/YY):**			
**Which order of events occured:**	**Y/N**	**Who entered waste info? U=user; W=witness**	**Was label explicitly shown (i.e. label turned to allow for reading) to witness? Y/N**
**1) Handoff complete and recruited witness first**			
**2) Handoff complete and logged into pyxis first**			
**3) Handoff NOT complete and recruited witness first**			
**4) Handoff NOT complete and logged into pyxis first**			
**5) Unrelated to handoff, pt was discharged from PACU**			
	**Time elapsed (min and sec)**	**Actual time of day (00:00 - 24:00)**	**Notes:**
**(If applicable) time between first witness request and transit time (i.e. time walking to pyxis)**			
**What was witness doing prior to recruitment?**	**Y/N**		
**1) EHR**			
**2) Direct patient care (e.g. at bedside)**			
**3) Indirect patient care (e.g. talking to another clinician/healthcare provider)**			
**4) Administrative tasks**			
**5) Non-clinical work**			
**6) Phone call (e.g.related to patient coordination, OR, etc.)**			
**7) Other:**			
**Pyxis Utilization:**	**Time Elapsed (min and sec)**	**Actual time of day (00:00 - 24:00)**	**Notes:**
**Log in time:**			
**Time from beginning to end of 1st medication waste**			
**Time from end of 1st med waste to end of 2nd medication waste**			
**Time from end of 2nd med waste to end of 3rd medication waste**			
**Log out time:**			
**(If applicable): Average pyxis time for each medication**			
**Which medications were wasted (write in order of wasting)?**			
**Was medication obtained for next case in the same session? If yes, how much total additional time was spent on this until log out time:**			
**Total cumulative time of wasting-related processes (= time from 1st to last waste):**			

**Total time in pyxis (=total time from log in to log out):**			
**Total waste pyxis time + transit time (total waste time + transit time w/ witness)**			
**Total time (transit + log into pyxis + waste + obtaining Rx + log out pyxis)**			
**Who entered waste into Pyxis:**	a) User entered waste information into pyxis<br>b) Witness entered waste information into pyxis<br>c) Mixed User/Witness entering waste information into pyxis		
**Other:**			
**Task interruption(s) during wasting:**			
**Additional time/motion not accounted for:**			
**Miscellaneous:**			

Supplemental Table 1. Case Report Form used for TMA data collection. This is the finalized case report form used after completing the pilot week of the study. Notes collected by the observing researcher (Cu) for each CS disposal event were documented using the above table.

Handoff	***"Handoff"*** was defined as the process by which the anesthesia team, usually composed of 2 clinicians (MD with a CRNA or resident physician), gave report to the respective PACU nurse taking over the care of a surgical patient. In most cases, if there was CS to be disposed of, the CS custodian disposed of medication either at the end of handoff or they would leave during handoff to dispose the CS while their partner gave report. At the SFVAMC, there is no standardization of when or how CS disposal should take place. However, site specific policies require that clinicians must dispose of medications within two hours of case completion.
Recruitment time	***"Recruitment time"*** described the time elapsed from the first announcement by a CS disposer to either the study coordinator or a witness that they needed assistance with a CS disposal procedure.
Transit time	***"Transit time"*** included the time traveled by the CS custodian to physically walk to the ADC as soon as an announcement to dispose was made. In most cases, recruitment and transit time overlapped as clinicians simultaneously walked towards the ADC and searched for an available witness. Therefore, the overlap in transit and recruitment time made it difficult to precisely parse the time difference between each process.
Total disposal time	***"Total disposal time"*** begins when the CS custodian first interacts with the ADC and ends when the user logs out of the device. A witness may or may not have been present for the entirety of this time depending on if they were before or after a CS custodian logged into the ADC.
Waste time	***"Waste time"*** describes a specific action within the broader category of disposals where residual CS from a patient case is appropriately documented into the ADC and is fully expelled into the appropriate waste bin adjacent to the ADC.
Return time	***"Return time"*** is a mutually exclusive event from wasting where a medication vial remained unopened and unused and is ultimately returned to the ADC. Both wastes and returns require a witness and their respective biometrics to confirm that the CS custodian followed appropriate disposal protocols. Overall, total disposal time includes, logging in to the ADC, searching for patient information and entering medication name, volume, and dose into the ADC for wastes and/or returns. Disposal time excluded the time required to obtain medications for future cases.
Total cumulative time	The ***"total cumulative time"*** captures the entirety of the wasting process from the first announcement that a CS disposal needed to

	occur to logging out of the ADC. This includes the time required to recruit a witness, the length of time required to travel the physical distance to the pyxis machine, the time spent interacting with the pyxis machine, the complete disposal of medication, and, if applicable, obtaining medications for future cases. Medications observed being wasted included: hydromorphone, midazolam, fentanyl, remifentanil, sufentanil, and ketamine. This process is visually described by Figure 2.

Supplemental Table 2: TMA terms glossary and definitions
Terms to describe components of CS disposal workflows were required to further differentiate different orders of events and their durations as they occurred. These were developed following 1 week of pilot time and motion analyses and iterated to a final case form.

Abbreviation: PACU=post-anesthesia care unit; CS=controlled substances; SFVAMC= San Francisco Veterans Affairs Medical Center; ADC=automated dispensing cabinet; TMA=time and motion analysis

Date: ____________________________
Provider Role (circle): RN / CRNA / MD / Resident MD

PART 1.

Note: You are welcome to skip any questions that make you uncomfortable. All answers will remain anonymous. Please how strongly you agree with the following statements where **1 = Extremely disagree and 7 = Extremely agree.**							
	**1**	**2**	**3**	**4**	**5**	**6**	**7**
Diversion could occur with our current (Pyxis-based) system							
Diversion will be identified with our current system							
Diversion is avoided with our current system							
The current process of detecting and surveilling controlled substance disposal is useful							
The current process of detecting and surveilling controlled substance disposal is labor-intensive							
The current process of detecting and surveilling controlled substance disposal is time-consuming							
Existing wasting processes are adequate to prevent discrepancies from occurring							
I am satisfied with the current wasting system							
The disposal system should incorporate direct testing of syringe or bag contents (e.g. through Raman Spectroscopy)							
Controlled substance discrepancies during disposal should be used as a predictor of diversion							
The security camera present in our PACU facilitates diversion monitoring							
The security camera present in our PACU is distracting							
The security camera present in our PACU invades my privacy							
I am comfortable with a camera being present and watching controlled substance disposals							

PART 2.

Note: You are welcome to skip any questions that make you uncomfortable. All answers will remain anonymous. **Please indicate the percentage range that you believe most agrees with each statement.**	
	**On a scale of 0-100%, please write your response below:**
What percentage of wasting events at your local institution do you believe are being diverted?	
What percentage of wasting events at your local institution do you believe witnesses read the label?	
What percentage of wasting events at your local institution do you believe witnesses read the volume of fluid in the syringe?	
What percentage of wasting events at an individual hospital do you believe are being diverted nationally (i.e. at other hospitals)?	

PART 3.

**The following questions are self-identification questions.** You are welcome to skip any questions that make you uncomfortable. All answers will remain anonymous.	
**Sex assigned at birth:**	▪ Male<br>▪ Female<br>▪ Prefer not to answer
**Sex assigned at birth:**	▪ Male<br>▪ Female<br>▪ Transgender<br>▪ Nonbinary<br>▪ Other: ________________<br>▪ Prefer not to answer
**Years of practice since obtaining degree:**	▪ 0-5 years<br>▪ 5-10 years<br>▪ 10-15 years<br>▪ 15+ years
**Age:**	▪ 20-34<br>▪ 35-49<br>▪ 50-64<br>▪ 65+
**Race (select all that apply):**	▪ White<br>▪ Black or African American

Note: You are welcome to skip any questions that make you uncomfortable. All answers will remain anonymous. **Please indicate the percentage range that you believe most agrees with each statement.**	
	▪ Middle Eastern or North African<br>▪ American Indian or Alaska Native<br>▪ Asian<br>▪ Native Hawaiian or Other Pacific Islander<br>▪ Other: ___________________
**Ethnicity (select all that apply):**	▪ Hispanic or Latino<br>▪ Not Hispanic or Latino<br>▪ Other: ___________________

Supplemental Table 3: Survey Questions Administered. The structure of Part 1 of the survey grouped questions by topic. We formatted our survey to have negative options to the left and positive options to the right with the value of 1 = extremely disagree, 2 = disagree, 3 = somewhat disagree, 4 = neither disagree or agree, 5 = somewhat agree, 6 = agree, 7 = extremely agree. This structure was based on the findings from Lewis[1] who found that there were less response errors with using numeric responses (as opposed to nominal labels) arranged with magnitude of agreement increasing from left to right.

Part 2 of the survey involved free response, and Part 3 asked for demographic information of the respondent.

Characteristics of CS Disposal Events	
**Order of events for wasting (pathways / phenotypes of wasting)**	N = 55
**Pathway 1:** Handoff completed and recruited witness first	28 (50.9%)
**Pathway 2:** Handoff completed and logged into pyxis first	10 (18.2%)
**Pathway 3:** Handoff NOT complete and recruited witness first	6 (10.9%)
**Pathway 4:** Handoff NOT complete and logged into pyxis first	3 (5.5%)
**Pathway 5:** Patient discharged from the PACU	8 (14.5%)
**Which combination of providers wasted medications?**	N = 55
MD & RN	7 (12.8%)
RN & RN	7 (12.8%)
CRNA & RN	14 (25.5%)
MD & CRNA	7 (12.8%)
MD & MD & SRNA	1 (1.8%)
MD & MD & CRNA	1 (1.8%)
CRNA & CRNA	2 (3.6%)
Resident MD & MD	11 (20%)
Resident MD & Resident MD	1 (1.8%)
Resident MD & RN	3 (5.5%)
Resident MD & MD & RN	1 (1.8%)
**What was the witness doing prior to recruitment?**	N = 55
Electronic health record	5 (9.1%)
Direct patient care*	28 (50.9%)
Indirect patient care**	3 (5.5%)
Administrative Tasks	13 (23.6%)
Non-clinical work	6 (10.9%)
**Events Observed**	N = 55
Wastes only (including obtaining medications for next case)	42 (76.4%)
Number of events that included obtaining medications for the next case	3
Returns only	7 (12.7%)
Mixed waste and returns	6 (10.9%)
Number of events that included obtaining medications for the next case	3

**Total Number of Medications Wasted*** **	69
Average	1.4
SD	0.57

Supplemental Table 4: Characteristics of CS Disposal Events. All values are rounded to the tenth decimal point. RN specifically refers to PACU nurses; MD identifies an attending physician; Resident MD identifies a physician in training; CRNA is a nurse anesthetist and SRNA is a student nurse anesthetist (who often lacked direct ADC credentials and needed the support of other clinicians to dispose)

* Direct patient care (e.g. a provider directly caring for a patient at their bedside or immediately completing patient handoff)

** Indirect patient care (e.g. talking to another clinician about patient care)

*** Medications wasted included: hydromorphone, midazolam, fentanyl, remifentanil, sufentanil, and ketamine

Abbreviations: PACU=post-anesthesia care unit; CS=controlled substances; ADC=automated dispensing cabinet; CRNA=certified nurse anesthetist; SRNA=student nurse anesthetist; SD=standard deviation

Times Observed Related to Disposal Processes					
	**Number of entries (N) ***	**Average time (sec)**	**SD (sec)**	**Median (sec)**	**Interquartile range (sec)**
Average **transit time** (includes time of re-cruiting witness and physical walk to pyxis)	51	32.2	81.3	10.8	13.4
Time to **waste 1st medication** (includes time entering information in pyxis)	49	39.8	46.7	30.9	27.7
Time to **waste subsequent medications** (i.e. 2nd and 3rd medication waste)	17	23.0	19.0	15.2	6.5
Time to **waste only** (regardless of the num-ber of total medications wasted per login event and includes searching information in pyxis)	49	47.8	34.4	35.2	33.7
Average time of **medication return** ONLY (excludes time to waste medications)	13	38.6	23.4	35.0	21.0
**Total disposal time in pyxis** (includes wastes and returns; excludes time to obtain medications for next case)	55	52.0	36.2	40.6	37.4
**Total cumulative time** (describes transit time and all subsequent events until logout of Pyxis; includes time to obtain medica-tions for next case)	55	88.1	92.7	52.0	53.2

Supplemental Table 5. All recorded times related to CS disposal.

Four transit and recruitment processes were not documented due events transpiring before the researcher could observe transit time and login at pyxis. For the above calculations, the total cumulative time for wastes and returns included events where wastes and returns occurred for a single pyxis login attempt. Unless otherwise specified, wasted medications were syringes. The majority of wasting events consisted of 1 medication and only rarely were there 2nd and 3rd medication disposals.

**Of the 55 total events, there were 49 recorded events with wasting and 6 events with returns only.*

Abbreviations: PACU=post-anesthesia care unit; CS=controlled substances; ADC=automated dispensing cabinet; CRNA=certified nurse anesthetist; SRNA=student nurse anesthetist; SD=standard deviation

**Survey Participant Demographics** (Total 28 Survey Responses)	
	**N (%)**
**Age:**	
20-34 years old	8 (28.6)
35-49 years old	10 (35.7)
50-64 years old	7 (25.0)
65+ years old	2 (7.1)
No Response	1 (3.6)
**Sex Assigned at Birth:**	
Male	14 (50)
Female	12 (42.9)
Other*/No response	2 (7.2)
**Self-Identified Gender:**	
Male	14 (50)
Female	12 (42.9)
Other*/No Response	2 (7.1)
**Years of Practice Since Obtaining Degree:**	9 (32.1)
0-5 years	5 (17.9)
5-10 years	3 (10.7)
10-15 years	2 (7.1)
15+ years	9 (32.2)
**Race:**	
White	9 (32.1)
Asian	10 (35.7)
Other*/No Response	9 (32.8)
**Ethnicity:**	
Not Hispanic or Latino	19 (67.9)
Hispanic or Latino	2 (7.1)
No response	7 (25.0)

Supplemental Table 6: Survey Participant Demographics. The survey was distributed to attending MDs, resident MDs, nurses, CRNA, and student nurse anesthetists. Participation in the survey was voluntary, and participants could choose if they did not want to their demographic information collected. To ensure anonymity, sex, gender, races and ethnicities that were low were listed as “Other” and grouped.

Survey Responses<br>(Total of 28 survey responses)<br><br>1 = Extremely Disagree, 2 = Disagree, 3 = Somewhat Agree, 4 = Neither Disagree or Agree, 5 = Somewhat Agree, 6 = Agree, and 7 = Extremely Agree										
**Question:**	**1 N (%)**	**2 N (%)**	**3 N (%)**	**4 N (%)**	**5 N (%)**	**6 N (%)**	**7 N (%)**	**No response N (%)**	**Median**	**Mode**
**Diversion could occur with our current (pyxis-based) system**	1 (3.6)	0 (0)	2 (7.1)	5 (17.9)	10 (35.7)	5 (17.9)	5 (17.9)	0 (0)	5	5
**Diversion will be identified with our current system**	2 (7.1)	5 (17.9)	4 (14.3)	5 (17.9)	7 (25.0)	1 (3.6)	2 (7.1)	2 (7.1)	4	5
**Diversion is avoided with our current system**	3(10.7)	3 (10.7)	3 (10.7)	11 (38.3)	5 (17.9)	2 (7.1)	0 (0)	1 (3.6)	4	4
**Existing wasting processes are adequate to prevent discrepancies from occurring**	3 (10.7)	3 (10.7)	6 (21.4)	7 (25.0)	4 (14.3)	4 (14.3)	0 (0)	1 (3.6)	4	4
**I am satisfied with the current wasting system**	2 (7.1)	3 (10.7)	2 (7.1)	7 (25.0)	9 (32.1)	2 (7.1)	2 (7.1)	1 (3.6)	4	5
**The disposal system should incorporate direct testing of syringe or bag contents (e.g. through Raman Spectroscopy)**	1 (3.6)	3 (10.7)	4 (14.3)	6 (21.4)	3 (10.7)	4 (14.3)	6 (21.4)	1 (3.6)	4	4
**Controlled substance discrepancies during disposal should be used as a predictor of diversion**	1 (3.6)	2 (7.1)	6 (21.4)	13 (46.4)	3 (10.7)	0 (0)	2 (7.1)	1 (3.6)	4	4
**The current process of detecting and surveilling controlled substance disposal is useful**	3 (10.7)	3 (10.7)	3 (10.7)	10 (35.7)	4 (14.3)	4 (14.3)	0 (0)	1 (3.6)	4	4
**The current process of detecting and surveilling controlled substance disposal is labor-intensive**	0 (0)	3 (10.7)	3 (10.7)	6 (21.4)	9 (32.1)	3 (10.7)	2 (7.1)	2 (7.1)	5	5
**The current process of detecting and surveilling controlled substance disposal is time-consuming**	0 (0)	2 (7.1)	4 (14.3)	2 (7.1)	10 (25.7)	6 (21.4)	3 (10.7)	1 (3.6)	5	5
**The security camera present in our PACU**	2 (7.1)	1 (3.6)	7 (25.0)	2 (7.1)	13 (46.4)	2 (7.1)	1 (3.6)	0 (0)	5	5

**facilitates diversion monitoring**										
**The security camera present in our PACU is distracting**	8 (28.6)	7 (25.0)	4 (14.3)	6 (21.4)	2 (7.1)	0 (0)	0 (0)	1 (3.6)	2	1
**The security camera present in our PACU invades my privacy**	6 (21.4)	5 (17.9)	3 (10.7)	7 (25.0)	4 (14.3)	2 (7.1)	1 (3.6)	0 (0)	3.5	4
**I am comfortable with a camera being present and watching controlled substance disposals**	1 (3.6)	3 (10.7)	1 (3.6)	4 (14.3)	8 (28.6)	5 (17.9)	6 (21.4)	0 (0)	5	5

Supplemental Table 7. Survey Responses. Exact number of responses for questions with Likert scale answer options are listed in this table. Percentages of responses per question is provided, along with median and mode of chosen Likert responses. 28 total surveys were collected. Participation was voluntary, and participants were informed that they could skip any questions listed.

Abbreviations: PACU=post-anesthesia care unit; CS=controlled substances; ADC=automated dispensing cabinet; CRNA=certified nurse anesthetist; SRNA=student nurse anesthetist; SD=standard deviation

Free Response Survey Questions and Responses (28 Total Survey Responses)					
	Number of Responses (N)	Median	IQR (Q1,Q3)	No response (N)	Unsure (N)
What percentage of wasting events at your local institution do you believe witnesses read the LABEL?	26	78%	0.5, 0.9	2	0
What percentage of wasting events at your local institution do you believe witnesses read the VOLUME?	27	75%	0.4, 0.9	1	0
What percentage of wasting events at an individual hospital do you believe are being diverted nationally (i.e. at other hospitals)?	20	25%	0.5, 0.35	6	2

Supplemental Table 8. Free response questions and responses. All responses were voluntary and did not require participants to provide an answer if they were uncomfortable.

Abbreviations: IQR = interquartile range. Q1 = quartile 1. Q3 = quartile 3.

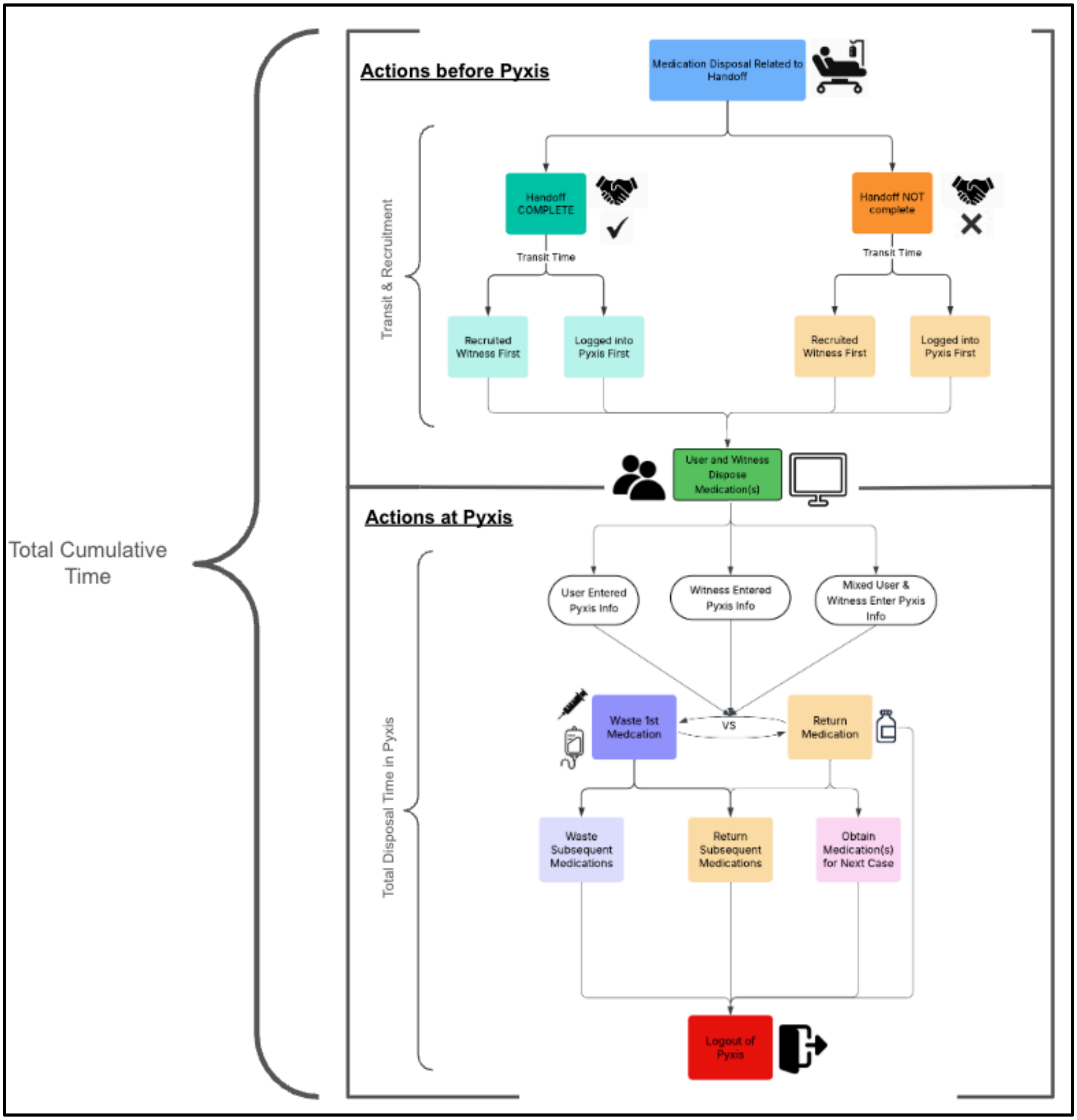

Supplemental Figure 1. Overall disposal processes at the SFVAMC. ***Total disposal time*** in pyxis includes wastes and returns but excludes time to obtain medications for future cases. ***Time to waste subsequent medications*** includes cases where a total of 2 to 3 medications were wasted. ***Total cumulative time*** describes the time from the initial declaration that a clinician needs to dispose of medications until they logged out of pyxis.
Abbreviations: SFVAMC=San Francisco Veterans Affairs Medical Center

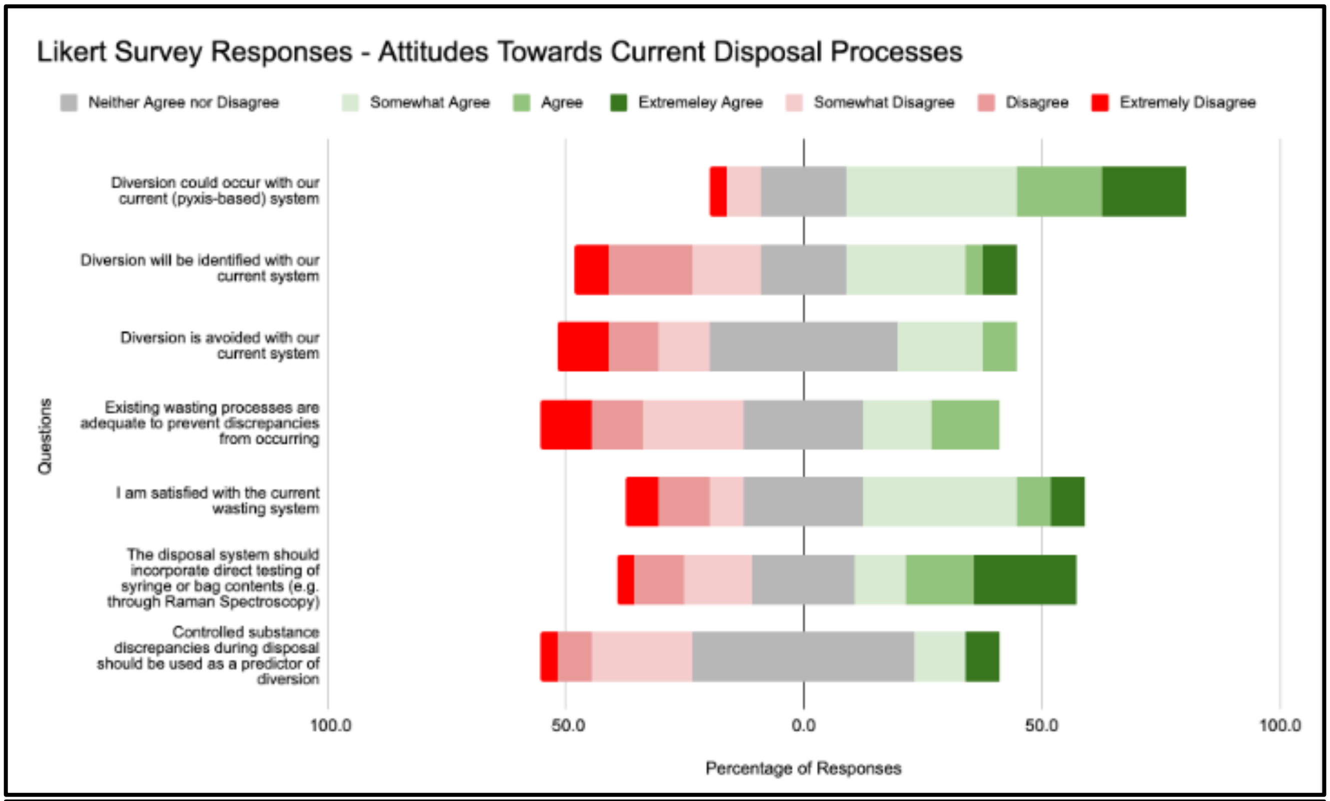

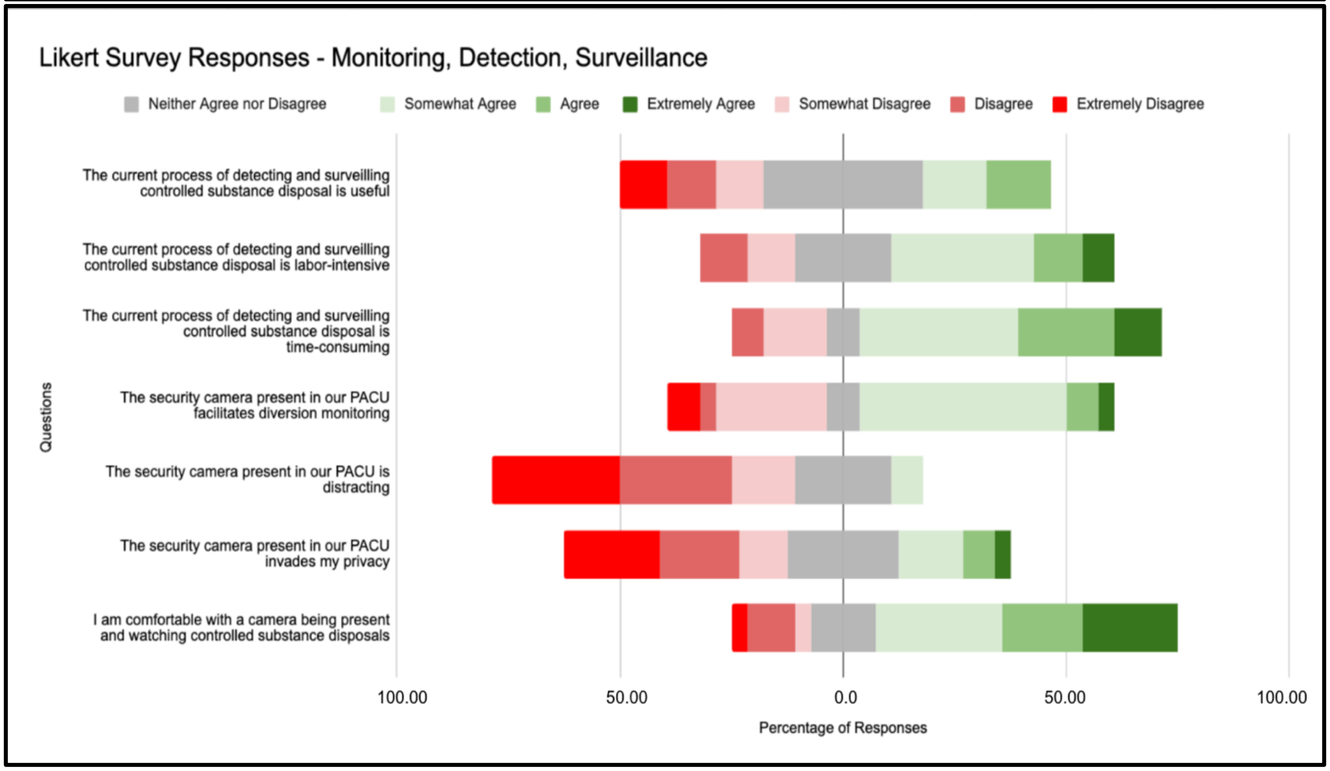

Supplemental **Figure 2. Dual-axis charts of survey responses regarding attitudes towards current disposal processes, monitoring, detection and CS surveillance.** All responses were voluntary and did not require participants to provide an answer if they were uncomfortable.

Specific response counts for each question can be found in Supplemental Table 8. Percentage distributions of responses are represented in the charts above.

Abbreviations: CS=controlled substances; sec=seconds; SD=standard deviation; ADC=automated dispensing cabinet

STROBE Statement—Checklist of items that should be included in reports of ***cohort studies***[5]

	Item No	Recommendation	Page No
**Title and abstract**	1	(*a*) Indicate the study's design with a commonly used term in the title or the abstract	1-3
		(*b*) Provide in the abstract an informative and balanced summary of what was done and what was found	1-3
**Introduction**			
Background/rationale	2	Explain the scientific background and rationale for the investigation being reported	4-5
Objectives	3	State specific objectives, including any prespecified hypotheses	4-5
**Methods**			
Study design	4	Present key elements of study design early in the paper	4-6
Setting	5	Describe the setting, locations, and relevant dates, including periods of recruitment, exposure, follow-up, and data collection	4
Participants	6	(*a*) Give the eligibility criteria, and the sources and methods of selection of participants. Describe methods of follow-up	4
		(*b*) For matched studies, give matching criteria and number of exposed and unexposed	
Variables	7	Clearly define all outcomes, exposures, predictors, potential confounders, and effect modifiers. Give diagnostic criteria, if applicable	5-6
Data sources/ measurement	8*	For each variable of interest, give sources of data and details of methods of assessment (measurement). Describe comparability of assessment methods if there is more than one group	4-6
Bias	9	Describe any efforts to address potential sources of bias	4-6, 10
Study size	10	Explain how the study size was arrived at	5
Quantitative variables	11	Explain how quantitative variables were handled in the analyses. If applicable, describe which groupings were chosen and why	4-6
Statistical methods	12	(*a*) Describe all statistical methods, including those used to control for confounding	4-6
		(*b*) Describe any methods used to examine subgroups and interactions	
		(*c*) Explain how missing data were addressed	
		(*d*) If applicable, explain how loss to follow-up was addressed	
		(*e*) Describe any sensitivity analyses	
**Results**			
Participants	13*	(a) Report numbers of individuals at each stage of study—eg numbers potentially eligible, examined for eligibility, confirmed eligible, included in the study, completing follow-up, and analysed	7-8
		(b) Give reasons for non-participation at each stage	
		(c) Consider use of a flow diagram	
Descriptive data	14*	(a) Give characteristics of study participants (eg demographic, clinical, social) and information on exposures and potential confounders	7-8
		(b) Indicate number of participants with missing data for each variable of interest	

		(c) Summarise follow-up time (eg, average and total amount)	
Outcome data	15*	Report numbers of outcome events or summary measures over time	7-8
Main results	16	(*a*) Give unadjusted estimates and, if applicable, confounder-adjusted estimates and their precision (eg, 95% confidence interval). Make clear which confounders were adjusted for and why they were included<br>(*b*) Report category boundaries when continuous variables were categorized<br>(*c*) If relevant, consider translating estimates of relative risk into absolute risk for a meaningful time period	7-8
Other analyses	17	Report other analyses done—eg analyses of subgroups and interactions, and sensitivity analyses	7-8
**Discussion**			
Key results	18	Summarise key results with reference to study objectives	8-10
Limitations	19	Discuss limitations of the study, taking into account sources of potential bias or imprecision. Discuss both direction and magnitude of any potential bias	8-10
Interpretation	20	Give a cautious overall interpretation of results considering objectives, limitations, multiplicity of analyses, results from similar studies, and other relevant evidence	8-10
Generalisability	21	Discuss the generalisability (external validity) of the study results	8-10
**Other information**			
Funding	22	Give the source of funding and the role of the funders for the present study and, if applicable, for the original study on which the present article is based	11
